# Subsequent chemotherapy reverses acquired tyrosine kinase inhibitor resistance and restores response to tyrosine kinase inhibitor in advanced non-small-cell lung cancer

**DOI:** 10.1186/1471-2407-11-90

**Published:** 2011-03-02

**Authors:** Renhua Guo, Xiaofen Chen, Tongshan Wang, Zhiyuan Zhang, Jin Sun, Yongqian Shu

**Affiliations:** 1 Department of Medical Oncology, the First Affiliated Hospital of Nanjing Medical University, Nanjing, Jiangsu 210029, PR China

## Abstract

**Background:**

Patients with advanced or metastatic non-small cell lung cancer (NSCLC) can develop acquired resistance to epidermal growth factor receptor tyrosine kinase inhibitors (TKIs) erlotinib and gefitinib. Here, we report the successful treatment with alternating chemotherapy and TKIs of two cases of advanced NSCLC who developed resistance to TKI.

**Case presentation:**

Two patients with advanced or metastatic NSCLC were treated with palliative chemotherapy followed by erlotinib/gefitinib. When TKI therapy failed, two cycles of chemotherapy were provided, which were followed by re-challenge with erlotinib or gefitinib.

**Conclusion:**

NSCLC patients with acquired TKI resistance should be managed aggressively whenever possible. Subsequent chemotherapy and target treatment is one of the reasonable choices for those with an initial dramatic clinical response with erlotinib/gefitinib treatment. Further studies are warranted to substantiate the association of erlotinib /gefitinib treatment with the efficacy of NSCLC patients with acquired TKI failure.

## Background

Lung cancer is a leading cause of cancer-related death throughout the world and its death toll reached 7.4 million or approximately 13% of all deaths worldwide in 2004. Early diagnosis of lung cancer is difficult due to the insidious nature of symptoms and most patients have progressed to an advanced stage at the time of diagnosis. Multiple studies have clearly shown that chemotherapy is important in the palliative care of advanced non-small cell lung cancer (NSCLC) even compared with the best supportive treatment. Chemotherapy as the main treatment approach for advanced lung cancer can improve patient survival and their quality of life [1].

Erlotinib and gefitinib, as oral epidermal growth factor receptor (EGFR) tyrosine kinase inhibitors (TKIs), are commonly used as second- or third-line drugs [2,3], and are sometimes also used in first-line therapy for advanced or metastatic NSCLC [4,5]. Despite an initial dramatic response, most patients treated with these two agents will eventually develop progressive disease. Few reports have reported on treatment options after acquired TKI failure. In the current report, we present two cases of advanced NSCLC. One patient received and benefited from gefitinib, and the other from erlotinib after repeated cycles of palliative chemotherapy and targeted therapy. The two patients are still in good conditions and alive 3-4 years after diagnosis with advanced lung cancer.

## Case presentation

### Case one

**Figure 1:**
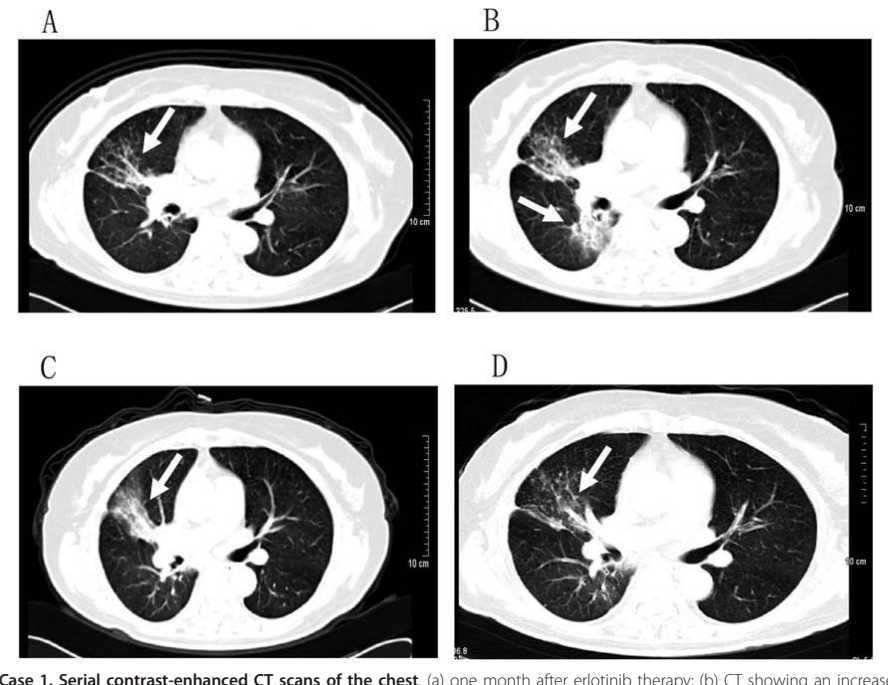
**Case 1. Serial contrast-enhanced CT scans of the chest**. (a) one month after erlotinib therapy; (b) CT showing an increase in size of the lung nodule after 12 months of erlotinib therapy; (c) CT showing a stable disease of the lung nodule after two cycles of chemotherapy; (d) CT showing a shrinkage of the tumor in the right lower lobe after 4 weeks of erlotinib re-challenge.

In June 2007, a 70-year-old non-smoking female was seen at our hospital because of a 3-month history of progressive dysponea at rest. CT scan revealed a mass at the right lower lobe and moderate pleural effusion on the right side. Metastasis to right adrenal gland was observed on abdominal CT. There was no evidence of extra thoracic metastasis on brain MRI and bone ECT scans. Lung needle aspiration revealed adenocarcinoma and the patient was diagnosed with NSCLC stage IV. She started 4 cycles of systemic chemotherapy with cisplatin and gemcitabine in Jul 2007. CT scan showed a stable disease of the carcinoma and marked clinical improvement was noted as dyspnoea disappeared and the patient reported a general feeling of wellness. The patient then received two cycles of docetaxel for maintenance therapy. In the following six months, the patient was well without any evidence of local or systemic recurrence. In June 2008 a routine follow-up bone ECT and brain MRI revealed bone and brain metastatic lesions, and the patient commenced erlotinib (150 mg daily ), which she tolerated well and only experienced grade 1 skin rash without requiring dose adjustment. After 4 weeks of erlotinib, the patient showed complete response in her intracranial disease and a partial response in her lung disease. After 12 months of erlotinib therapy, tumor at the right lower lobe progressed, and two cycles of carboplatin and paclitaxel were administered. CT imaging confirmed stable disease in the right lower lobe tumor. However, the patient refused further cytotoxic chemotherapy because of severe treatment-related diarrhea. She was re-challenged with erlotinib (150 mg daily ) in October 2009, and experienced grade 3 skin rash without dose modification. Fortunately, she improved clinically with her right lower lobe tumor showing partial response after 4 weeks of erlotinib treatment and the treatment was continued for eleven more months(CT scans were shown in Figure 1).

### Case two

**Figure 2:**
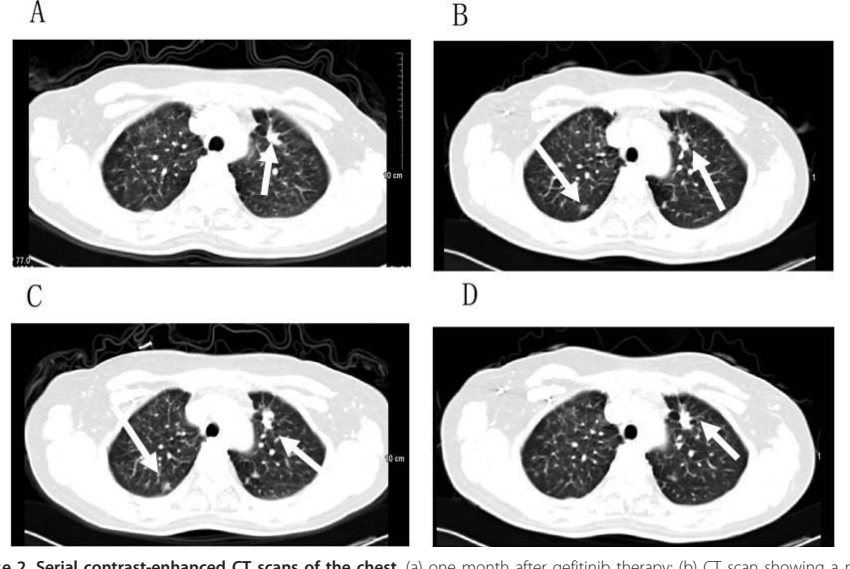
**Case 2. Serial contrast-enhanced CT scans of the chest**. (a) one month after gefitinib therapy; (b) CT scan showing a new lesion at the right lund after 29 months of gefitinib therapy; (c) CT showing a stable disease of the lung nodule after two cycles of chemotherapy; (d) CT scan showing a marked regression of the tumor in the right lung, and stable disease of the tumor in the left lung after one month of gefitinib re-challenge.

In September 2006, a 50-year-old non-smoking female was seen by us because of a 4-month history of non-productive cough and shortness of breath. A mass in the left upper lobe and multiple lesions in both lungs were observed on chest CT, and multiple bone involvements were found on ECT. Lung fine-needle aspiration showed the presence of adenocarcinoma. She was initially treated with 4 cycles of systemic chemotherapy with cisplatin and gemcitabine. CT scans demonstrated progressive disease. She was administered two cycles of docetaxel, and a progressive disease was noted in the lung disease. Therefore, she commenced gefitinib (250 mg daily) as third-line therapy in May 2007. She tolerated the regimen well and only experienced grade 1 diarrhea. After 4 weeks of gefitinib, the patient showed partial response in her lung disease. After 29 months of gefitinib, the patient developed brain metastases and the tumor also spread to the right lung. Brain radiotherapy (25 Gy/five fractions) was administered followed by two cycles of carboplatin and paclitaxel. Chemotherapy, however, was terminated because of severe hematological toxicity. She was re-challenged with gefitinib (250 mg daily) in Nov 2009 and experienced grade 1 skin rash and diarrhea. After 4 weeks of gefitinib, a CT scan showed a marked regression in the right lung tumor and intracranial disease, Stable disease was noted in the left lung tumor. So far, the patient continued gefitinib treatment(CT scans were shown in Figure 2).

## Discussion

Systemic chemotherapy has played an important role in the palliative care of advanced NSCLC. Both quantitative and qualitative benefits have been noted with platinum-based 2-drug chemotherapeutic regimens in advanced NSCLC patients with a good performance status [6,7].

Patients with advanced NSCLC, however, eventually relapse or become refractory to first-line treatment. Acceptable toxicity and improved quality of life are especially important for those patients in second-line therapy. Docetaxel and pemetrexed, have demonstrated efficacy and been approved as second-line therapy for patients with advanced or metastatic NSCLC [8-10]. Meanwhile, for patients who do not respond to first-line chemotherapy or who tolerate it poorly, an EGFR inhibitor may be the preferred choice in second line therapy.

Erlotinib and gefitinib are EGFR TKIs that suppress intracellular signaling pathways, which normally promote cell growth and proliferation [11]. They have no cumulative hematologic toxicities, allowing for a longer duration of treatment. In the current study, erlotinib was administered as the second-line drug in one case and gefitinib as the third-line drug in the other case, and a long-term disease control was obtained from EGFR TKI treatment. Most patients treated with these agents, however, had progressive disease even after showing an initial dramatic response. Then, the strategy of treatment in the next step is of importance.

Currently, there is no clear evidence suggesting an optimal treatment strategy for patients with acquired TKI failure. Some trials are investigating the role of anticancer therapies in the third- or fourth-line setting after EGFR TKI failure. An irreversible TKI targeting the EGFR, HER2, and vascular endothelial growth factor receptor was evaluated in the third- and fourth-line settings after EGFR TKI failure [12]. A small molecule Met inhibitor combined with an EGFR TKI has proven to be a reasonable strategy to overcome erlotinib-resistant T790M NSCLC [13]. Combining an insulin-like growth factor inhibitor with erlotinib to reverse resistance to erlotinib is also under investigation [14]. However, most of these treatments are not clinically available. Subsequent chemotherapy was investigated after acquired TKI resistance and improved survival outcome in advanced NSCLC [15].

Our current two cases highlighted good responses to erlotinib/ gefitinib, and had obtained long-term stable disease with the second or third-line therapy initially. When the patients developed acquired resistance to EGFR TKIs, two cycles of a taxane-based chemotherapy was selected, after which erlotinib/gefitinib was administered again, which yielded more than ten months progression-free survival. Restored sensitivity to EGFR-TKIs after chemotherapy may be due to the following mechanisms: first, the proportion of sensitive and resistant cells in the tumor could have been modified by the treatment as chemotherapy may have killed erlotinib/gefitinib resistant cells, and erlotinib/gefitinib sensitive cells have become dominant. Secondly, chemotherapy may induce novel genetic mutations in EGFR or other unknown associated genes that regulated resistance to TKI.

Recently, some Asian cases have been described in whom chemotherapy can restore gefitinib efficacy after gefitinib resistance [16-18], indicating that drug resistance may be reversible [19,20]. Gefitinib has been reported to modulate the chemotherapy resistance of NSCLC [21]. Several cancer cell line studies already highlighted the importance of drug interaction between TKI and taxane [22-24]. Thus, EGFR-TKIs and chemotherapy may result in a sequence-dependent tumor regression.

Based on the data herein, it is suggested that acquired TKI resistance be managed aggressively whenever possible. Subsequent chemotherapy and target treatment are one of the reasonable choices. Since TKI resistance becomes increasingly important in the treatment of NSCLC, our results provide promising insight for future clinical practice and future investigations. Erlotinib/gefitinib could be re-considered for those NSCLC patients who had already benefited from prior erlotinib/gefitinib treatment. Further studies are warranted to substantiate the association of erlotinib /gefitinib treatment with the efficacy of NSCLC patients with acquired TKI failure.

## Consent

Written informed consent was obtained from the two patients for publication of this case report and any accompanying images. A copy of the written consent is available for review by the Editor-in-Chief of this journal.

## Competing interests

The authors declare that they have no competing interests.

## Authors' contributions

RHG and XFC analyzed the data and wrote the manuscript. TSW, ZYZ, JS made substantial contributions in data acquisition. YQS participated in study design and coordinate among the authors. All authors read and approved the final manuscript.

## Pre-publication history

The pre-publication history for this paper can be accessed here:

http://www.biomedcentral.com/1471-2407/11/90/prepub
